# Synthesis of MgAC-Fe_3_O_4_/TiO_2_ hybrid nanocomposites via sol-gel chemistry for water treatment by photo-Fenton and photocatalytic reactions

**DOI:** 10.1038/s41598-019-48398-5

**Published:** 2019-08-14

**Authors:** Vu Khac Hoang Bui, Duckshin Park, Tuyet Nhung Pham, Yejin An, Jin Seok Choi, Hyun Uk Lee, Oh-Hyeok Kwon, Ju-Young Moon, Ki-Tae Kim, Young-Chul Lee

**Affiliations:** 1 Department of BioNano Technology, Gachon University, 1342 Seongnamdaero, Sujeong-gu, Seongnam-si, Gyeonggi-do, 13120 Republic of Korea; 2 Korea Railroad Research Institute (KRRI), 176 Cheoldobakmulkwan-ro, Uiwang-si, 16150 Gyeonggi-do, Republic of Korea; 3 Department of Environmental Engineering, Seoul National University of Science and Technology, 232 Gongneung-ro, Nowon-gu, Seoul, 01811 Republic of Korea; 4 Analysis Center for Research Advancement, Korea Advanced Institute of Science and Technology (KAIST), Yuseong-gu, Daejeon, 34141 Republic of Korea; 5 Advanced Nano-surface Research Group, Korea Basic Science Institute (KBSI), Daejeon, 34133 Republic of Korea; 6 Department Beauty Design Management, Hansung University, 116 Samseongyoro-16gil, Seoul, 02876 Republic of Korea

**Keywords:** Photocatalysis, Photocatalysis

## Abstract

MgAC-Fe_3_O_4_/TiO_2_ hybrid nanocomposites were synthesized in different ratios of MgAC-Fe_3_O_4_ and TiO_2_ precursor. X-ray diffraction (XRD), scanning electron microscopy (SEM), transmission electron microscopy (TEM), X-ray fluorescence spectrometry (XRF), electron spin resonance spectrometry (ESR), Brunauer-Emmett-Teller (BET), photoluminescence (PL), and UV photoelectron spectroscopy (UPS) were used to characterize the nanocomposites. The increase of MgAC-Fe_3_O_4_, in the hybrid nanocomposites’ core-shell structure, led to the decrease of anatase TiO_2_ peaks, thus reducing the photo-Fenton and photocatalytic activities. According to the obtained data, MgAC-Fe_3_O_4_ [0.05 g]/TiO_2_ showed the best photo-Fenton and photocatalytic activities, having removed ~93% of MB (photo-Fenton reaction) and ~80% of phenol (photocatalytic reaction) after 20 and 80 mins, respectively. On the pilot scale (30 L), MgAC-Fe_3_O_4_ [0.05 g]/TiO_2_ was completely removed after 27 and 30 hours by the photo-Fenton and photocatalytic activities, respectively. The synergistic effect gained from the combined photo-Fenton and photocatalytic activities of Fe_3_O_4_ and TiO_2_, respectively, was credited for the performances of the MgAC-Fe_3_O_4_/TiO_2_ hybrid nanocomposites.

## Introduction

Advanced oxidation processes (AOPs) including O_3_/H_2_O_2_, UV/O_3_, UV/H_2_O_2_, H_2_O_2_/Fe^2+^ and UV/TiO_2_ have been utilized for removal of toxic organic compounds in water/waste water, air, and soil. AOPs produce hydroxyl radicals that contain powerful oxidants capable of oxidizing various organic compounds with one or many double bonds^1^. Recently, the potential of photocatalytic materials has been extended to other applications such as photocorrosion inhibition, solar water splitting by combined with different materials^2–4^. Among AOPs materials, photocatalysis with TiO_2_ nanoparticles (NPs) under UV/Visible light has attracted keen interest from the time of its first discovery by Frank and Bard^5^, specifically due to its high oxidative power and chemical stability^6–8^. However, the limitation of TiO_2_ NPs is their low quantum efficiency of photo-generated electron-hole pairs caused by a high and rapid recombination rate^9^. For example, Degussa P25, a well-known commercial product, can reduce only 14% of phenol at 365 nm in water^10^. Another major disadvantage of TiO_2_ NPs is that they cannot be recycled post-reaction. Leakages of photocatalytic materials to aqueous solution, moreover, can lead to secondary pollution. These disadvantages can be overcome by coating TiO_2_ NPs on the surfaces of magnetic components such as Fe_3_O_4_ NPs, which can be easily collected from solutions under magnetic fields^11^.

Fe_3_O_4_ NPs have attracted interest due to their considerable magnetic behavior and strong pin polarization^12^. Many methods of Fe_3_O_4_-TiO_2_ composite synthesis, such as sol-gel, co-precipitation, hydrothermal, sonochemical, and templates routes, have been reported in the literature^12,13^. Incorporation of Fe_3_O_4_ NPs into a TiO_2_ matrix can block NP aggregation and improve the durability of catalysts^14,15^. However, due to the small band gap of Fe_3_O_4_ NPs (0.1 eV), Fe_3_O_4_-TiO_2_ composites will increase the rate of electron-hole pairs recombination, with the result that photocatalysis is usually unchanged or even diminished relative to pure TiO_2_ NPs^12^. To overcome this problem, Zheng *et al*. demonstrated that special structures such as core-shell microspheres in Fe_3_O_4_-TiO_2_ composites can delay the recombination of photo-induced electrons^12^; other researchers have used noble metal (Au or Ag) or rare elements (e.g. Eu) as electron traps to enhance electron-hole separation and facilitate electron excitation by creating a local hole in the electrical field^13,16,17^. In contrast, He *et al*., after preparing Fe_3_O_4_-TiO_2_ core-shell NPs, indicated that Fe^3+^ released from Fe_3_O_4_ can be doped into TiO_2_ NPs to decrease electron-hole pair recombination and thus increase the photocatalytic performance of Fe_3_O_4_-TiO_2_ core-shell NPs under visible light^18^. One remarkable report in this research field is that of Sun *et al*., who found that a small number Fe_3_O_4_ NPs loaded onto TiO_2_ NPs (Fe/TiO_2_ ratio: 1/200) could enhance the degradation of organic dye (Reactive Brilliant Red X3B). They attributed the improved photocatalytic performance of Fe_3_O_4_-TiO_2_ to the synergistic contribution of the photocatalytic and Fenton reactions in the composite^19^.

2-D materials have been attracted and extended their applications due to their unique properties^20^. Photocatalytic materials have also been developed based on 2-D materials such as graphene^21^.

From its first introduction by Mann *et al*., magnesium aminoclay (MgAC), which is also another types of 2-D materials, has attracted interest in its propylamine functionalities, structures, and high dispersity in water^22–24^. Use of MgAC’s high adsorption utility for heavy metal and organic dye removal has been reported^25^. Besides being utilized as a single agent, MgAC has been conjugated with other materials for environmental-treatment purposes. For example, MgAC has been coated with nZVI for removal of perfluorinated compounds^26^ and chromium^27^.

In previous work, we conjugated MgAC with TiO_2_ NPs and Fe_3_O_4_ NPs by different methods for environmental-treatment^28,29^ and microalgae-harvesting purposes^30,31^. The presence of MgAC in composites was demonstrated to improve the photocatalytic behavior of pure TiO_2_ NPs^28^ as well as the photo-Fenton behavior of Fe_3_O_4_ NPs^29^. Based on these successful preliminary results, in the present study, we synthesized MgAC-Fe_3_O_4_/TiO_2_ hybrid composites in order to exploit the advantages of both Fe_3_O_4_ and TiO_2_ in environmental-treatment applications.

## Results and Discussion

### Photo-Fenton performances of MgAC-Fe_3_O_4_/TiO_2_ hybrid nanocomposites on batch scale

**Figure 1:**
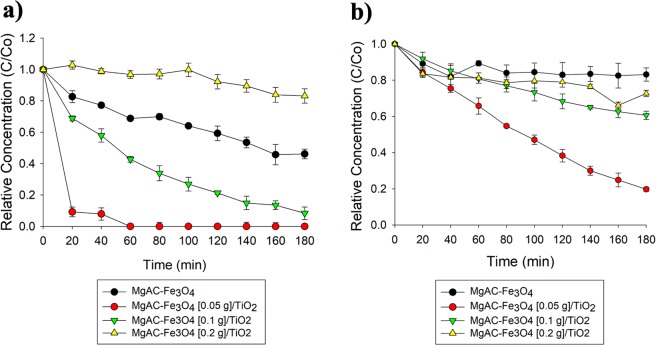
Photo-Fenton (MB, **a**) and photocatalytic performances (phenol, **b**) of MgAC-Fe_3_O_4_/TiO_2_ hybrid nanocomposites on batch scale.

Among different MgAC-Fe_3_O_4_/TiO_2_ hybrid nanocomposites on the batch scale, the MgAC-Fe_3_O_4_ [0.05 g]/TiO_2_ sample showed the best photo-Fenton performance, more than ~93% of MB having been removed after 20 min at a constant rate of ~0.1175 min^−1^; this was ~10 and ~100 times higher than the performances of MgAC-Fe_3_O_4_ [0.1 g]/TiO_2_ and MgAC-Fe_3_O_4_ [0.2 g]/TiO_2_, which removed ~92 and ~17% of MB from aqueous solution after 180 min of reaction, respectively (Fig. 1a and Table 1).

**Table 1:** Degradation rates of MgAC-Fe_3_O_4_/TiO_2_ hybrid nanocomposites by photo-Fenton and photocatalytic reaction on batch scale.

Sample	Photo-Fenton constant rate against MB 10 ppm (min^−1^)	Photocatalytic constant rate against phenol 5 ppm (min^−1^)
**MgAC**-**Fe**_**3**_**O**_**4**_ [**0**.**05** **g**]/**TiO**_**2**_	**0**.**1175**	**0**.**0039**
MgAC-Fe_3_O_4_ [0.1 g]/TiO_2_	0.0130	0.0012
MgAC-Fe_3_O_4_ [0.2 g]/TiO_2_	0.0011	0.0006

We chose the MgAC-Fe_3_O_4_ [0.05 g]/TiO_2_ sample to investigate the photo-Fenton performance when inhibition agents such as humic acid (HA), SO_4_^2−^, and HCO_3_^−^ existed in the aqueous solution. Among these three inhibition agents, SO_4_^2−^ and HCO_3_^−^ had stronger inhibitory effects on the photo-Fenton reactions of the hybrid samples than HA, especially when these anionic and organic substances were presence at high concentrations in water (Fig. S2). The presence of HA, SO_4_^2−^, and HCO_3_^−^ can limit the photo-Fenton reaction of hybrid samples by reacting with generated reactive oxygen species (ROS)^32^.

### Photocatalytic performances of MgAC-Fe_3_O_4_/TiO_2_ hybrid nanocomposites on batch scale

Instead of MB, we chose phenol to investigate the photocatalytic performances of the MgAC-Fe_3_O_4_/TiO_2_ hybrid nanocomposites on the batch scale. Phenol is widely utilized in wastewater treatment within various industrial fields such as resins, petrochemicals, paints, textiles, oil refineries, foods, photographic chemicals, antioxidants and flavoring agents^33^. Phenol is a highly toxic pollutant that can cause environmental degradation and serious health problems in humans.

Among the samples, MgAC-Fe_3_O_4_ [0.05 g]/TiO_2_ showed the best photocatalytic activity, ~80% of phenol having been removed after 180 min at a constant rate of 0.0039 min^−1^; MgAC-Fe_3_O_4_ [0.1 g]/TiO_2_ and MgAC-Fe_3_O_4_ [0.2 g]/TiO_2_, by comparison, had removed only 40 and 27% of phenol, respectively (Fig. 1b and Table 1). The photocatalytic activities of the MgAC-Fe_3_O_4_/TiO_2_ hybrid nanocomposites were attributed to the synergistic contributions of the photocatalytic and Fenton reactions in the hybrid nanocomposites^19^. In previous studies, Sun *et al*. indicated that increased Fe_3_O_4_ NP number could lead to decreased photocatalytic activity. Excess loading of Fe_3_O_4_ NPs promotes cluster aggregation, reduces O_2_ adsorption, and thereby decreases the efficiency of interfacial charge transfer for pollutant degradation^19^.

After 5 times recycle, the MgAC-Fe_3_O_4_ [0.05 g]/TiO_2_ sample still remained ~80% of its initial removal efficiencies against phenol (Fig. S3). The deactivation of MgAC-Fe_3_O_4_ [0.05 g]/TiO_2_ could be explained by the accumulation of phenol intermediate products on actives sites of hybrid nanocomposites^34^. The presence of these intermediate products in the reactor after stopping photocatalytic reaction could be supported by results of TOC below^35^. The hybrid nanocomposites could not be regenerated by using simple washing methods^34^. In the future works, the more suitable regeneration methods should be considered to improve the recycling performances.

### Photo-fenton and photocatalytic mechanisms

The photocatalytic mechanism of MgAC-Fe_3_O_4_/TiO_2_ was attributed to the presence of anatase TiO_2_ in the samples, as follows:

$${{\rm{TiO}}}_{2}+hv\to {{\rm{TiO}}}_{2}({e}^{-}+{h}^{+})$$

$${h}^{+}+{{\rm{H}}}_{2}{\rm{O}}\to {H}^{+}+O{H}^{\bullet }$$

$${h}^{+}+{{\rm{OH}}}^{-}\to {{\rm{OH}}}^{\bullet }$$

$${e}^{-}+{O}_{2}\to {}^{\bullet }\,{{\rm{O}}}_{2}^{-}$$

$${{\rm{Fe}}}^{3+}+{{\rm{TiO}}}_{2}({{\rm{e}}}^{-}+{h}^{+})\to {{\rm{Fe}}}^{2+}+{{\rm{TiO}}}_{2}({h}^{+})$$

OH^•^ and ^•^O_2_^−^, which are produced through the above chain reactions, will degrade pollutant molecules via oxidation and a reduction reaction process, respectively. To investigate the contribution of these ROS to the photocatalytic activity of MgAC-Fe_3_O_4_/TiO_2_, different scavengers have been used: isopropanol for •OH, methanol for both h^+^ and •OH, and *p*-benzoquinone for ^•^O_2_^−^ ^36,37^. In our system, highly reactive •OH was the main actor in the degradation of the pollutant materials, rather than the valence band h^+^ and the conductive band e^−^ (Fig. S4)^36^.

On the other hand, the photo-Fenton activities of the MgAC-Fe_3_O_4_/TiO_2_ hybrid nanocomposites were extremely high, due to the presence of H_2_O_2_ in the reaction:

$${{\rm{Fe}}}^{2+}+{{\rm{H}}}_{2}{{\rm{O}}}_{2}\to {}^{\bullet }\,{\rm{OH}}+{{\rm{Fe}}}^{3+}+{{\rm{OH}}}^{-}$$

$${{\rm{H}}}_{2}{{\rm{O}}}_{2}+hv\to {}^{\bullet }\,{\rm{OH}}$$

As noted just above, pollutants are degraded mainly by ^•^OH, which is generated in a photo-Fenton-like process: direct photolysis of H_2_O_2_, and photocatalytic oxidation of adsorbed H_2_O through holes in the valence band of the TiO_2_ surface. Additionally, photo-induced electrons generated from TiO_2_ can cause reduction of Fe^3+^ to Fe^2+^ ^38^. The photocatalytic and photo-Fenton activities of MgAC-Fe_3_O_4_/TiO_2_ hybrid nanocomposites can be additionally supported by the presence of MgAC, which, with its high adsorption utility, can bring reactants to the surfaces of photo-Fenton agents^24,25^.

Photoluminescence spectra could be used to explain the photocatalytic mechanism^39^. From photoluminescence spectra (Fig. S5), it is clearly seen that, due to the presence of TiO_2_ in the hybrid composites, the separated electron and holes were kept longer in excited state than original MgAC-Fe_3_O_4_ samples^40,41^. These results also were used to support the photocatalytic mechanisms of MgAC-Fe_3_O_4_/TiO_2_ hybrid nanocomposites. In addition, MgAC-Fe_3_O_4_ [0.05 g]/TiO_2_ have the lowest photoluminescence intensity.

### Preliminary evaluation of photocatalytic and photo-Fenton performances of MgAC-Fe_3_O_4_/TiO_2_ hybrid nanocomposites on pilot scale

The photocatalytic activities of TiO_2_ NP materials have been widely investigated on the batch scale. However, for the purposes of industrial application, these materials need to be up-scaled to pilot-scale reactors. There are a variety of pilot reactors that have been introduced in the literature^26,27^. In this study, we used a design of pilot reactor that has been introduced in previous reports^28,29^. The design and photos of the pilot reactor are presented in Fig. S6.

**Figure 2:**
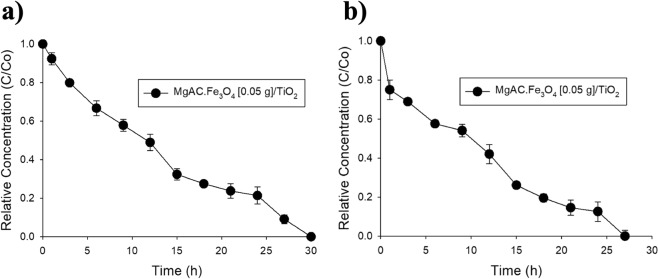
(**a**) Photocatalytic and (**b**) photo-Fenton performances of MgAC-Fe_3_O_4_ [0.05 g]/TiO_2_ hybrid nanocomposites on pilot scale.

Based on the batch-scale results discussed above, MgAC-Fe_3_O_4_ [0.05 g]/TiO_2_ was mass produced for testing of photocatalytic and photo-Fenton performances on the pilot scale. For photocatalytic performance testing, 30 g of MgAC-Fe_3_O_4_ [0.05 g]/TiO_2_ was added to the 30 L pilot reactor to obtain a dosage of 1 g/L (similarly to the batch-scale study). After 30 hours, phenol had been nearly completely removed from the aqueous solution at a constant rate of 0.032 (h^−1^) (Fig. 2a and Table 2). The extended reaction time might be attributable to the heaviness of the materials, which is quickly self-precipitated. However, this phenomenon makes MgAC-Fe_3_O_4_ [0.05 g]/TiO_2_ easily recoverable after the reaction. In this study, ~80% of the materials was recovered from the reactor by simply stopping the reactor and allowing self-precipitation to occur for 24 hours without any external force (Fig. S7). To shorten the reaction time, we added 50 mL of H_2_O_2_ to obtain ~15 ppm peroxide in the reactor while reducing the dosage of MgAC-Fe_3_O_4_ [0.05 g]/TiO_2_ from 1 g/L to 0.5 g/L. Under this photo-Fenton condition, phenol was completely removed after 27 hours at a constant rate of increase to 0.036 (h^−1^) (Fig. 2b and Table 2). It should be noted that in this paper, we present just the preliminary results; the optimal concentrations of photocatalytic materials and H_2_O_2_ should be discussed in future work investigating the effect of tap water on degradation rate. There is also a requirement for continual reactor upgrading to prevent quick sedimentation in the photocatalytic reaction and, thereby, improve the performance of MgAC-Fe_3_O_4_ [0.05 g]/TiO_2_ on the pilot scale.

**Table 2:** Phenol-degradation rates of MgAC-Fe_3_O_4_/TiO_2_ hybrid nanocomposites by photocatalytic and photo-Fenton reaction on pilot scale.

MgAC-Fe_3_O_4_ [0.05 g]/TiO_2_	Constant rate (h^−1^)
Photocatalytic	0.0032
Photo-Fenton	0.0036

For phenol of 500 ppm, after 48 h exposure, the LC_50_ was 6.39 mg/L (5.36–7.53, 95% CI). At the concentrations of 0, 2.5, 5, 7.5, 10, and 20 ppm, the mortality rates were 0, 15, 20, 50, 80, and 100%, respectively (Fig. S1b). Meanwhile, the treated samples showed a mortality rate of ~10% (Fig. S1c). We suspected that the remnant toxicity had come from the leakage of Fe^3+^ ions from the hybrid nanocomposites or the phenol intermediate products after the reactions. So, we conducted both ICP and TOC experiments to investigate the reason behind the toxicity of the treated samples. The ICP results showed that the leakage of iron ions after stoppage of the reaction was negligible (~60 ppb after treatment, lower than the standard 300 ppb for drinking water according to the WHO)^42^. However, the TOC experiments showed that the organic carbon concentration was still very high after the reaction (~50–55%); thus, the toxicity could be attributed to the residual toxic phenol intermediate products (Fig. S8). Therefore, it is necessary to extend the reaction until intermediate products are completely removed, not to the phenol concentration of zero^36^.

### Characterization of hybrid nanocomposites

**Figure 3:**
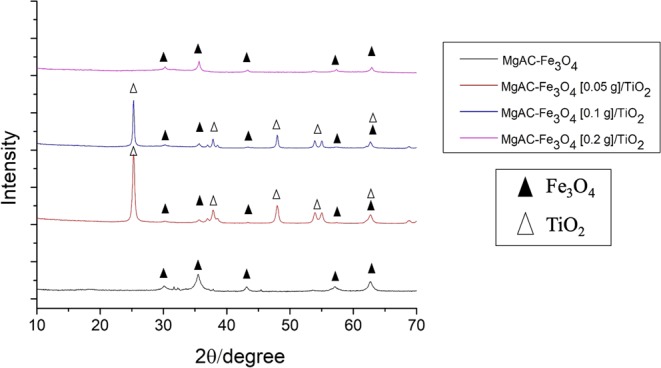
XRD patterns of MgAC-Fe_3_O_4_ and MgAC-Fe_3_O_4_/TiO_2_ hybrid nanocomposites.

The magnetic properties of the MgAC-Fe_3_O_4_/TiO_2_ hybrid nanocomposites were preserved after the synthesis process (Fig. S9). The XRD pattern showed that, for the MgAC-Fe_3_O_4_ sample, the peaks at 29.98°, 35.5°, 42.9°, 56.7°, and 62° belonged respectively to (200), (311), (400), (511), and (440) of Fe_3_O_4_ magnetite (JCDS 00-021-1272; JCDS: Joint Committee on Power Diffraction Standard)^28^; meanwhile, for MgAC-Fe_3_O_4_ [0.05 g]/TiO_2_ and MgAC-Fe_3_O_4_ [0.1 g]/TiO_2_, besides the reduced MgAC-Fe_3_O_4_ peaks, there were additional peaks at 25.5°, 37.8°, 48.3°, 54.2°, and 62.8° belonging respectively to the (101), (004), (200), (105), and (204) planes of the anatase phase (JCDS 00-064-0863, Fig. 3)^28^. It was apparent that, for MgAC-Fe_3_O_4_ [0.1 g]/TiO_2_, the high-intensity peaks of TiO_2_ were reduced, whereas those of MgAC-Fe_3_O_4_ [0.2 g]/TiO_2_ had totally disappeared. It could be concluded that the excess MgAC-Fe_3_O_4_ loading inhibited the growth of anatase TiO_2_ in the hybrid nanocomposites. XRD was double checked by conducting two measurement to confirm the phenomena (Fig. S10). These results were similar with Sun *et al*.^19^. Also, the XPS analysis showed that, for MgAC-Fe_3_O_4_ [0.05 g]/TiO_2_, besides the peaks at ~532, ~285, ~102, and ~52 eV belonging respectively to O 1 s, C1s, Si 2p, and Mg 2p (Fig. S11a)^27,43^, there were additional peaks at ~463 eV and ~457 eV that were attributed to the Ti 2p_1/2_ and Ti 2p_3/2_ of Ti^4+^ states of stoichiometric TiO_2_, respectively (Fig. S11b)^44^. The Fe 2p_1/2_ and Fe 2p_3/2_ peaks of Fe_3_O_4_ existed at ~723.9 and ~710.2 eV, respectively (Fig. S11c)^45^.

**Figure 4:**
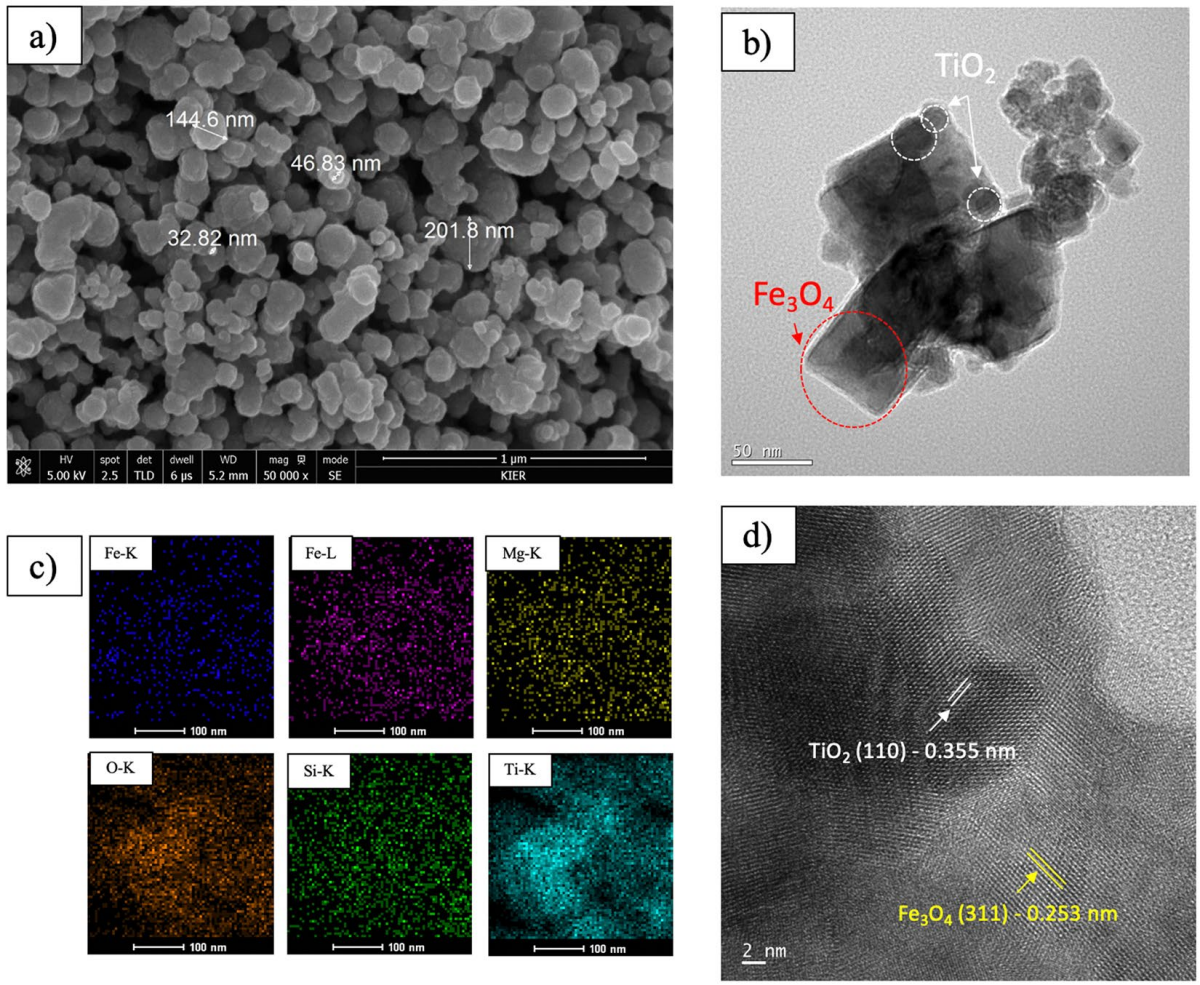
Morphology of MgAC-Fe_3_O_4_ [0.05 g]/TiO_2_ hybrid nanocomposite. (**a**) SEM, (**b**) TEM, (**c**) EDX mapping analysis, and (**d**) HRTEM.

**Figure 5:**
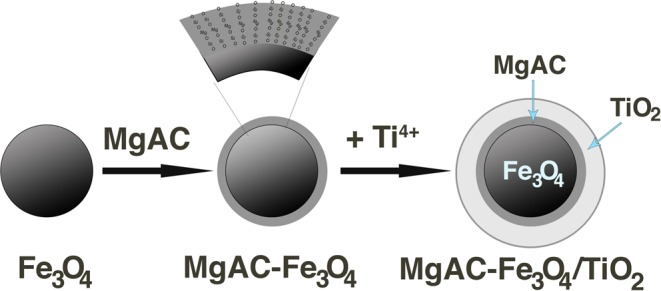
Scheme of process of MgAC-Fe_3_O_4_/TiO_2_ synthesis.

According to the SEM results, MgAC-Fe_3_O_4_ [0.05 g]/TiO_2_ had an aggregated form, with a diameter ranging between 32.82 and 201.80 nm (Fig. 4a). The morphology of MgAC-Fe_3_O_4_ [0.05 g]/TiO_2_ was further investigated by TEM and energy-dispersive X-ray mapping analysis (EDX). In the XRD, TEM, and EDX results, where MgAC-Fe_3_O_4_ was uniformly distributed in the TiO_2_ matrix, MgAC-Fe_3_O_4_/TiO_2_ showed a core-shell-like structure, MgAC-Fe_3_O_4_ playing the role as the core material and TiO_2_ that of the out-layer material (Fig. 4b,c). Lattice fringe spacing of 0.253 nm, belonging to the (311) plane of the Fe_3_O_4_ NPs, and 0.355 nm of TiO_2_ NPs in HR-TEM image confirmed the presence of these particles in the hybrid nanocomposites (Fig. 4d)^46,47^. From the XRF analysis results, the ratios between Fe_3_O_4_ and TiO_2_ were ~1:12, 1:5.5, and 1:3.5 for MgAC-Fe_3_O_4_ [0.05 g]/TiO_2_, MgAC-Fe_3_O_4_ [0.1 g]/TiO_2_, and MgAC-Fe_3_O_4_ [0.2 g]/TiO_2_, respectively (Table S1). XRF and XRD confirmed the effects of MgAC-Fe_3_O_4_ loading on the photocatalytic activities of the MgAC-Fe_3_O_4_/TiO_2_ hybrid nanocomposites. Sun (2018) indicated that a ratio between Fe and TiO_2_ of 1:200 could enhance the degradation of organic dye, increase the loading amount of Fe_3_O_4_ and, thus, decrease the photocalytic activity^19^. The amounts of MgO and SiO in the XRF analysis and of Mg, Si in the EDX mapping analysis were attributed to the presence of amorphous MgAC in the hybrid nanocomposites (Fig. 5c and Table S1).

As for the ESR spectrum, the two main signals at ~2.0 and ~4.3 could be assigned to different Fe(III) sites^48^. The broad signal at ~2.0 could be assigned to the interaction between iron (octahedral sites) and MgAC. This band could belong to the chain -O-Si-O-Fe-O-Si-O- from the reaction between the MgAC and Fe_3_O_4_ NPs^49^. The second signal at g ~4.3 corresponded to the strongly orthorhombic sites located on the surface or to the isolated Fe(III) ions dispersed in the silica matrix^50^. Additionally, the symmetric broad band at g ~2.0 confirmed the ferromagnetic properties of the MgAC-Fe_3_O_4_/TiO_2_ hybrid nanocomposites (Fig. S12)^49^. Based on the characteristic data, the suggested structure of MgAC-Fe_3_O_4_/TiO_2_ is provided in Fig. 5. In the synthesis of MgAC-Fe_3_O_4_, the presence of amino groups in the layered MgAC structure makes for high dispersibility in water^25^. The formation of the NH_3_^+^ and –OH groups on the surfaces of Fe_3_O_4_ NPs helps them to connect through strong electrostatic interaction. When the calcine process was carried out, besides the decomposition of amine groups on the surface material, there was evident formation of new bond bridges such as (-Fe-O-C_3_-Si-O-Mg-O-Si-O-C_3_-) within the architecture. (-C_3_-O-Si-Mg-O-Si-O-C_3_-) bridges within MgAC, for example, are very stable under harsh conditions. The large-interface surface area between Fe_3_O_4_ and MgAC facilitates formation of new bridges between Fe_3_O_4_ and MgAC at high temperature. This formation of new bridges suggests possible coordination of the O elements with the carbon elements and replacement of the O atoms in the NH_3_ groups^51^. Fe_3_O_4_ NPs are not easy to coat with TiO_2_ NPs layers, and so the presence of SiO_2_ could overcome this problem^52,53^. Moreover, the SiO_2_ layer formed in hybrid NPs can make space for the adsorption of polluted materials^53,54^. For synthesis of MgAC-Fe_3_O_4_/TiO_2_, adding TiO_2_ to the surfaces of synthetic material not only leads to increased photocatalytic activity but also forms a stable inter-connection. We offered some great bridges at the interface of the TiO_2_ NPs and Fe_3_O_4_, such as (-O-Ti-O-Fe-O-Si-O-Mg-O-Si-O-Fe-O-Ti-O-) or (-O-Ti-O-Si-O-Mg-O-Si-O-Ti-O-). Based on the synthetic condition, it was clearly seen that many O atoms existing on the material surface could have resulted in the development of these new bridges. Besides, Si atoms are more electronegative and less polarizable than Fe and Ti atoms; the effective positive charge on Fe and Ti is increased, and the effective negative charge on O is decreased. In other words, the electron density around Fe and Ti atoms is decreased and the shielding effect is weakened, which results in increased binding energy.

From the obtained UPS spectra (Fig. S13), the work functions were calculated to 2.98, 1.87, 2.1, 1.95, and 1.56 eV for MgAC-Fe_3_O_4_, MgAC-Fe_3_O_4_ [0.05 g]/TiO_2_, MgAC-Fe_3_O_4_ [0.1 g]/TiO_2_, MgAC-Fe_3_O_4_ [0.2 g]/TiO_2_, and MgAC-TiO_2_, respectively (Table S2). It was noted that MgAC-TiO_2_ was synthesized by using MgAC and titanium butoxide (TB) as precursors in ethanol media, as previously reported^28^. The decrease of work function could facilitate electron emission and narrow the energy band gap^55^. It was clearly seen that the work function of MgAC-Fe_3_O_4_ was lower than that of Fe_3_O_4_, which is around 3.7 eV in the literature^56^; this reduction of work function could be explained by the higher photo-Fenton activity of MgAC-Fe_3_O_4_ than that of commercial Fe_3_O_4_, as was demonstrated in a previous report^29^. The presence of TiO_2_ on the surface of MgAC-Fe_3_O_4_ continued to decrease the work function, the lowest number belonging to MgAC-Fe_3_O_4_ [0.05 g]/TiO_2_ (1.87 eV, optimal sample), which was yet larger than that of MgAC-TiO_2_ (1.56 eV), which showed photocatalytic activation under visible light activation^28^. It could be concluded that, in general, the photocatalytic activity of the MgAC-Fe_3_O_4_/TiO_2_ hybrid nanocomposite is higher than that of MgAC-Fe_3_O_4_ (which has only photo-Fenton activity) but lower than that of MgAC-TiO_2_ (which also has photocatalytic activity under visible light irradiation). The advantages of MgAC-Fe_3_O_4_/TiO_2_ relative to MgAC-TiO_2_ come from its recycling utility. The difference of photocatalytic activity between the two might come from their respective synthesis processes and material structures. In the case of MgAC-TiO_2_, nitrogen from the amino-functional groups of MgAC can be doped into the TiO_2_ structure via a calcination process, thus inducing photocatalytic activity under visible light^57^. However, in the case of MgAC-Fe_3_O_4_/TiO_2_ hybrid nanocomposites, the nitrogen element is removed by the process of the synthesis of MgAC-Fe_3_O_4_, as suggested above.

Additionally, the surface areas, pore sizes and pore volumes of the materials were investigated to characterize their surfaces. As can be seen in Table 3, the surface area, pore size and pore volume of the MgAC-Fe_3_O_4_ sample were the lowest (surface area: 34.790 m^2^/g; pore size: 3.639 nm; pore volume: 0.0356 cm^3^/g). In the literature, the adsorption capacity of Fe_3_O_4_ is lower than that of TiO_2_^19^. In the present study, the MgAC-TiO_2_ sample had the largest surface area, pore size and pore volume (surface area: 87.822 m^2^/g; pore size: 7.595 nm; pore volume: 0.1510 cm^3^/g). Surprisingly, for the MgAC-Fe_3_O_4_/TiO_2_ hybrid nanocomposites, when TiO_2_ existed on the surfaces of the MgAC-Fe_3_O_4_ particles, the surface area was not significantly changed (exception: MgAC-Fe_3_O_4_ [0.1 g]/TiO_2_), whereas the pore size and pore volume were increased. For instance, for MgAC-Fe_3_O_4_ [0.05 g]/TiO_2_ (the optimal sample), the pore size and pore volume were 9.4163 and 0.0834 cm^3^/g, 3 times larger than those of MgAC-Fe_3_O_4_. In general, even though the surface areas of MgAC-Fe_3_O_4_/TiO_2_ hybrid nanocomposites are not significantly changed, their absorption capacities, via the increased pore sizes and volumes, are higher than that of MgAC-Fe_3_O_4_.

**Table 3:** BET surface areas, pore sizes and pore volumes of samples in this study.

Sample	BET surface area (m^2^/g)	Pore size (nm)	Pore volume (cm^3^/g)
MgAC-Fe_3_O_4_	34.790	3.639	0.0356
MgAC-Fe_3_O_4_ [0.05 g]/TiO_2_	35.159	9.4163	0.0834
MgAC-Fe_3_O_4_ [0.1 g]/TiO_2_	70.244	6.652	0.1280
MgAC-Fe_3_O_4_ [0.2 g]/TiO_2_	31.44	10.629	0.0899
MgAC-TiO_2_	87.822	7.595	0.1510

## Discussion

MgAC-Fe_3_O_4_/TiO_2_ samples were synthesized via sol-gel methods. The core-shell structure with Fe_3_O_4_ as the core component, SiO_2_ as the middle layer and TiO_2_ as outer layer was suggested. Based on the laboratory scale, the cost of MgAC-Fe_3_O_4_/TiO_2_ materials has been calculated to around 1,479.80 USD/kg, similar to that of MgAC-TiO_2_ (1,476.24 USD/kg; Table S3), due to its higher production efficiency (1 g MgAC can produce only 1.08 g of MgAC-TiO_2_, whereas 1 g MgAC-Fe_3_O_4_ can produce 4.4 g of MgAC-Fe_3_O_4_ [0.05 g]/TiO_2_). Another advantage of MgAC-Fe_3_O_4_/TiO_2_ is its quick sedimentation, which enables ~80% of materials to be recovered after 24 hours of self-precipitation. Moreover, with their ferromagnetism was remained after the synthesis process, these hybrid nanocomposites show their recycling potential. However, the remnant toxicity of the treated sample against *Daphnia magna* indicated the presence of residual phenol intermediate products. The comparison of our study with some remarkable researches in the literature was briefly summarized in the Table 4.

**Table 4:** The comparison of our study with some remarkable researches in the literature.

	Preparation Method	Obtained material	Remarkable results
He *et al*.^18^	Homogenous precipitation	Core-shell Fe_3_O_4_-TiO_2_	• Obtained Fe_3_O_4_-TiO_2_ is non-toxic • Fe^3+^ could be doped into TiO_2_ and activate the photocatalytic activation under visible light activation
Sun *et al*.^19^	One-step calcination	Magnetic Fe_3_O_4_-TiO_2_	• The degradation of organic dye by Fe_3_O_4_-TiO_2_ (Fe/TiO_2_ ratio: 1/200) was enhanced compared to single Fe_3_O_4_ and TiO_2_ • The synergistic of Fe_3_O_4_ and TiO_2_ could be attributed to the high photocatalytic activity
Zheng *et al*.^12^	Liquid phase deposition	Waxberry-like microsphere Fe_3_O_4_-TiO_2_	• Diameter: ~500 nm • Shell thickness: ~10–20 nm • Remove 40% of MB (10 ppm) after 60 mins under Xenon lamp (300 W) • Could be recycled after photocatalytic reaction
Stefan *et al*.^13^	Ultrasound assisted sol-gel	Fe_3_O_4_-TiO_2_: Eu nanocomposite	• Increase of Eu doping decrease the formation of FeTiO_3_ • Large surface area and mesoporous strcuture • Remove 85% of RhB (1.0 × 10^−5^ mol/L) dye after 3 h unter visible light irradiation (400 W halogen lamp)
Alzahani^53^	Sol-gel	Core shell Fe_3_O_4_/SiO_2_/TiO_2_	• Under UV light, the photocatalytic performance was higher than commercial TiO_2_
**This study**	**Sol**-**gel**	**MgAC**-**Fe**_**3**_**O**_**4**_/**TiO**_**2**_	• **The synergistic of Fe**_**3**_**O**_**4**_ **and TiO**_**2**_ **could be attributed to the performances of MgAC**-**Fe**_**3**_**O**_**4**_/**TiO**_**2**_ • **Core**-**shell structure with Fe**_**3**_**O**_**4**_ **as core and TiO**_**2**_ **at outer layer is suggested**. • **However**, **the photocatalytic under visible light should be activated in the near future**

For industrial-scale application purposes, the toxicity potential of MgAC-Fe_3_O_4_/TiO_2_ as well as its photo-Fenton and photocatalytic mechanisms should be more thoroughly investigated. Additionally, it is necessary to find a way to induce the photocatalytic activity of such hybrid nanocomposites under visible light.

## Methods

### Materials

(3-aminopropyl)trielthoxysilane (APTES; ≥98%, 221.37 g/mol), iron (III) chloride hexahydrate (III) (FeCl_3_ · 6H_2_O; 97%) and titanium butoxide (TB; 97%) were obtained from Sigma-Aldrich (St. Louis, MO, USA). Magnesium chloride hexahydrate (MgCl_2_ · 6H_2_O; 98%) was purchased from Junsei Chemical (Tokyo, Japan). Ethanol (18 L, 95%) was acquired from Samchun Pure Chemicals (Pyungtack, Korea). NaOH (pellet, 97%) was purchased from Daejung Chemical & Metals (Siheung, Korea). Distilled-deionized water (DI; resistance: >18 mΩ) was employed in all of the experiments.

### Synthesis of magnesium aminoclay (MgAC)

For preparation of MgAC, 1.68 g of MgCl_2_ · 6H_2_O was dissolved in 40 mL of ethanol. Then, 2 mL of 3-aminopropyltrielthoxylane (APTES) was added and stirred for 8 hours to form a white suspension. The resultant suspension was then centrifuged and washed with ethanol (3 × 50 mL) before being dried at 40 °C and ground into powder^58^.

### Synthesis of magnesium aminoclay-iron oxide (MgAC-Fe_3_O_4_) nanocomposites

A total of 0.7 g of MgAC was mixed with 3 g of FeCl_3_ · 6H_2_O in 40 mL of DI water, to which mixture 10 mL of NaOH 10 M was added. The solution was stirred for 12 hours and then centrifuged and washed with DI water (3 × 50 mL) and dried at 60 °C to form a brown solid. The brown products were ground into powder and calcinated at 500 °C for 3 hours in a furnace (FU-100TG, Samheung Energy, Korea) under 4% H_2_/Ar (flow rate: 0.15 L/min) to produce MgAC-Fe_3_O_4_ nanocomposites^29^.

### Synthesis of magnesium aminoclay-iron oxide/TiO_2_ (MgAC-Fe_3_O_4_/TiO_2_) hybrid nanocomposites

In order to prepare MgAC-Fe_3_O_4_/TiO_2_ hybrid nanocomposites, respectively 0.05 g, 0.1 g and 0.2 g of MgAC-Fe_3_O_4_ were mixed with 1 mL of TB in 40 mL EtOH, to each of which mixtures 0.25 µL of DI water was slowly added; the resultant solutions were denoted MgAC-Fe_3_O_4_ [0.05 g]/TiO_2_, MgAC-Fe_3_O_4_ [0.1 g]/TiO_2_, and MgAC-Fe_3_O_4_ [0.2 g]/TiO_2_, respectively. Each solution was then stirred for at least 12 hours, washed with ethanol (3 × 50 mL) and dried to form a grey solid. The resultant products were then ground and consequently calcinated at 350 °C for 3 hours under air in a muffle furnace to produce MgAC-Fe_3_O_4_/TiO_2_ hybrid nanocomposites^28^.

### Photo-fenton performances of MgAC-Fe_3_O_4_/TiO_2_ hybrid nanocomposites on batch scale

A total of 0.1 g of MgAC-Fe_3_O_4_/TiO_2_ hybrid nanocomposites was loaded into 100 mL of methylene blue (MB; Sigma-Aldrich, St. Louis, MO, USA) at a concentration of 10 ppm. After obtainment of equilibrium adsorption, 1 mL of H_2_O_2_ (35%) was added, and a 365 nm (24 W) UV light source was turned on. One (1) mL of treated water was withdrawn after an interval of 20 min, and the MB concentration was observed under UV-Vis spectroscopy (Cary 50-UV Vis Spectrophotometers, Varian Inc., USA) at a wavelength of 664 nm^28^.

The degradation rate of the organic compounds was determined by the equation

$$-\,\frac{[C]}{dt}=k[{C}_{o}]$$

where C is the concentration of MB at time (t), C_o_ is the initial MB concentration, and k is the pseudo-first-order rate constant (min^−1^)^19^.

### Photocatalytic performances of MgAC-Fe_3_O_4_/TiO_2_ hybrid nanocomposites on batch scale

For evaluation of the photocatalytic performances of MgAC-Fe_3_O_4_/TiO_2_ hybrid nanocomposites, 0.1 g of MgAC-Fe_3_O_4_/TiO_2_ hybrid nanocomposites was loaded into a petri dish of 100 mL of phenol at 5 ppm (≥99%; Sigma-Aldrich, St. Louis, MO, USA). After obtainment of equilibrium adsorption, a 365 nm wavelength UV light source (~610 µW/cm^2^) was turned on. Interval samples were withdrawn after each 20 min, and the remaining organic compounds were detected by high-performance liquid chromatography (for phenol; HPLC, Waters Alliance 2695 Separations Module equipped with Waters 2487 Dual λ Absorbance Detector; Waters, Milford, MA, USA) under the mobile phase of water and acetonitrile in a ratio of 40:60 (v/v) with a flow rate of 1 mL/min^59^. For detection of phenol, UV absorption was performed at 270 nm.

The recycle usage experiments were conducted to check the stability of materials. After photocatalyst materials were separated from degraded solution, they were washed with DI water and ethanol, then dried in the oven at 60 °C for 12 hours. Then the materials is ready for another photocatalytic experiments. This method was repeated for 5 times^53^.

### Preliminary evaluation of photocatalytic and photo-Fenton performances of MgAC-Fe_3_O_4_/TiO_2_ hybrid nanocomposites on pilot scale

The photocatalytic performances of the MgAC-Fe_3_O_4_/TiO_2_ hybrid nanocomposites were tested using the systems introduced in a previous report^28^. Briefly, MgAC-Fe_3_O_4_/TiO_2_ hybrid nanocomposites were loaded into a reactor (90 cm (width) × 30 cm (depth) × 60 cm (height)) containing 30 L of tap water contaminated with phenol at 3 ppm and stirred with 3 stirrers (GGM speed control motor, Korea) at 145 rpm. After obtainment of equilibrium adsorption, 18 × 365 nm wavelength UV lamps (light intensity: ~610 µW/cm^2^, 65 cm × 3 cm) were turn on. After an interval of 3 hours, 40 mL of treated water was withdrawn, and the remaining concentration of phenol was determined by HPLC. For evaluation of the photo-Fenton performances of the MgAC-Fe_3_O_4_/TiO_2_ hybrid nanocomposites, H_2_O_2_ was supplied after obtainment of equilibrium adsorption.

For an ecotoxicity test, *Daphnia magna* was incubated in a 16 h light/8 h darkness cycle at 21 ± 1 °C with M4 medium prepared according to OECD Test Guideline 202 (OECD, 2014)^60^. The culture medium was replaced daily, and *Chlorella* was fed once a day. The *Daphnia* test was carried out according to OECD Test Guideline 202, and young daphanids aged less than 24 hours were collected and exposed to the test materials for 48 hours after *Chlorella* feeding for 2 hours. In the case of 500 ppm phenol feedstock, 5 daphanids were exposed to 25 mL of 0, 2.5, 5, 7.5, 10, and 20 ppm phenol to determine the LC_50_ (Fig. S1a). Ecotoxicity tests of photo-Fenton- and photocatalytic-treated samples against *Daphnia magna* also were performed. The toxicity of the photo-Fenton and photocatalytic samples also were investigated, first, via inductive coupled plasma atomic emission spectroscopy (ICP-AES; Optima 7300 DV, Pelkin Elmer, USA) for leakage of iron ions and, second, via total organic carbon (TOC, Vario TOC Cube, Elementar, Germany) for the presence of intermediate products, after the reaction.

### Characterization

The crystallography of the MgAC-Fe_3_O_4_/TiO_2_ hybrid nanocomposites was examined using a Rigaku D/max-2500 (18 kW, Japan) incorporating a θ/θ goniometer equipped with a 40 kV, 30 mA CuKα radiation generator. The morphology of the MgAC-Fe_3_O_4_/TiO_2_ hybrid nanocomposites was investigated by scanning electron microscopy (SEM; SEM-4700, Japan) and transmission electron microscopy (TEM; JEM-2100F, JEOL Ltd., USA). X-ray fluorescence spectrometry (XRF; MiniPal 2, PANanalytical, Almelo, Netherlands) was employed to identify the elemental compositions of as-prepared samples. The surface areas, pore sizes and pore volumes of the materials were investigated by Brunauer-Emmett-Teller (BET; Micromeritics ASAP 2010, USA)^25^. The photoluminescence (PL) spectra were obtained to investigate the electron-hole fate of the semiconductor (DUT-260, Core Bio System, Korea)^40^. The magnetic properties of the MgAC-Fe_3_O_4_/TiO_2_ hybrid nanocomposites were examined by electron spin resonance spectrometry (ESR; EMXplus/ELEXYS E580, Bruker, USA).

Next, for investigation of the optical properties of the materials, UV photoelectron spectroscopy (UPS; Axis Ultra DLD, Japan) with He I line (21.2 eV) UV source was applied. The work functions of the materials were calculated by the equation

$$WF=hv-|{E}_{cutoff}-\,{E}_{F}|$$

where *hv* is the incident energy (21.2 eV), *E*_*cutoff*_ is the secondary electron cutoff energy, and *E*_*f*_ is the Fermi energy^55^.

## Supplementary information

Supplementary Information

## Data Availability

The datasets generated and analysed during the current study are available from the corresponding author on reasonable request.
