# Author Correction: Synthesis of MgAC-Fe_3_O_4_/TiO_2_ hybrid nanocomposites via sol-gel chemistry for water treatment by photo-Fenton and photocatalytic reactions

**DOI:** 10.1038/s41598-019-53341-9

**Published:** 2019-11-13

**Authors:** Vu Khac Hoang Bui, Duckshin Park, Tuyet Nhung Pham, Yejin An, Jin Seok Choi, Hyun Uk Lee, Oh-Hyeok Kwon, Ju-Young Moon, Ki-Tae Kim, Young-Chul Lee

**Affiliations:** 1 Department of BioNano Technology, Gachon University, 1342 Seongnamdaero, Sujeong-gu, Seongnam-si, Gyeonggi-do, 13120 Republic of Korea; 2 Korea Railroad Research Institute (KRRI), 176 Cheoldobakmulkwan-ro, Uiwang-si, 16150 Gyeonggi-do, Republic of Korea; 3 Department of Environmental Engineering, Seoul National University of Science and Technology, 232 Gongneung-ro, Nowon-gu, Seoul, 01811 Republic of Korea; 4 Analysis Center for Research Advancement, Korea Advanced Institute of Science and Technology (KAIST), Yuseong-gu, Daejeon, 34141 Republic of Korea; 5 Advanced Nano-surface Research Group, Korea Basic Science Institute (KBSI), Daejeon, 34133 Republic of Korea; 6 Department Beauty Design Management, Hansung University, 116 Samseongyoro-16gil, Seoul, 02876 Republic of Korea

Correction to: *Scientific Reports* 10.1038/s41598-019-48398-5, published online 14 August 2019

The original version of this Article contained a typographical error in the spelling of the author Hyun Uk Lee, which was incorrectly given as Hyun-Uk Lee. This has now been corrected in the PDF and HTML versions of the Article, and in the accompanying Supplementary Information file.

