# Supplementary material for: Synthesis of MgAC-Fe3O4/TiO2 hybrid nanocomposites via sol-gel chemistry for water treatment by photo-Fenton and photocatalytic reactions
Source: Sci Rep. 2019 Aug 14;9:11855. doi: 10.1038/s41598-019-48398-5 (PMC6694146; doi:10.1038/s41598-019-48398-5)
Supplement: Supplementary file 1 — Supplementary Information [file 41598_2019_48398_MOESM1_ESM.pdf]

Synthesis of MgAC-Fe_3O_4/TiO_2 hybrid nanocomposites via sol-gel chemistry for water treatment by photo-Fenton and photocatalytic reactions

Supporting Information

Vu Khac Hoang Bui[1], Duckshin Park[2], Tuyet Nhung Pham[1], Yejin An[3], Jin Seok Choi[4], Hyun Uk Lee[5], Oh-Hyeok Kwon[6], Ju-Young Moon[6], Ki-Tae Kim[3*], and Young-Chul Lee[1*]

[1] Department of BioNano Technology, Gachon University, 1342 Seongnamdaero, Sujeong-gu, Seongnam-si, Gyeonggi-do 13120, Republic of Korea; hoangvu210190@gmail.com; nhungpham240694@gmail.com

[2] Korea Railroad Research Institute (KRRI), 176 Cheoldobakmulkwan-ro, Uiwang-si 16150, Gyeonggi-do, Republic of Korea; dspark@krri.re.kr

[3] Department of Environmental Engineering, Seoul National University of Science and Technology, 232 Gongneung-ro, Nowon-gu, Seoul 01811, Republic of Korea; yejin33@seoultech.ac.kr

[4] Analysis Center for Research Advancement, Korea Advanced Institute of Science and Technology (KAIST), Yuseong-gu, Daejeon 34141, Republic of Korea; ffband@kaist.ac.kr

[5] Advanced Nano-surface Research Group, Korea Basic Science Institute (KBSI), Daejeon 34133, Republic of Korea; leeho@kbsi.re.kr

[6] Department Beauty Design Management, Hansung University, 116 Samseongyoro-16gil, Seoul 02876, Republic of Korea; beauty67@hansung.ac.kr: bora7033@naver.com

*Correspondence:

Prof. Ki-Tae Kim; Email: ktkim@seoultech.ac.kr; Tel: +82-2-970-6642 (K.-T.K.); Fax: +82-2-971-5776 (K.-T.K.)

Prof. Young-Chul Lee; Email: dreamdbs@gachon.ac.kr; Tel: +82-31-750-8751 (Y.-C. L.); Fax: +82-31-750-4748 (Y.-C. L.)

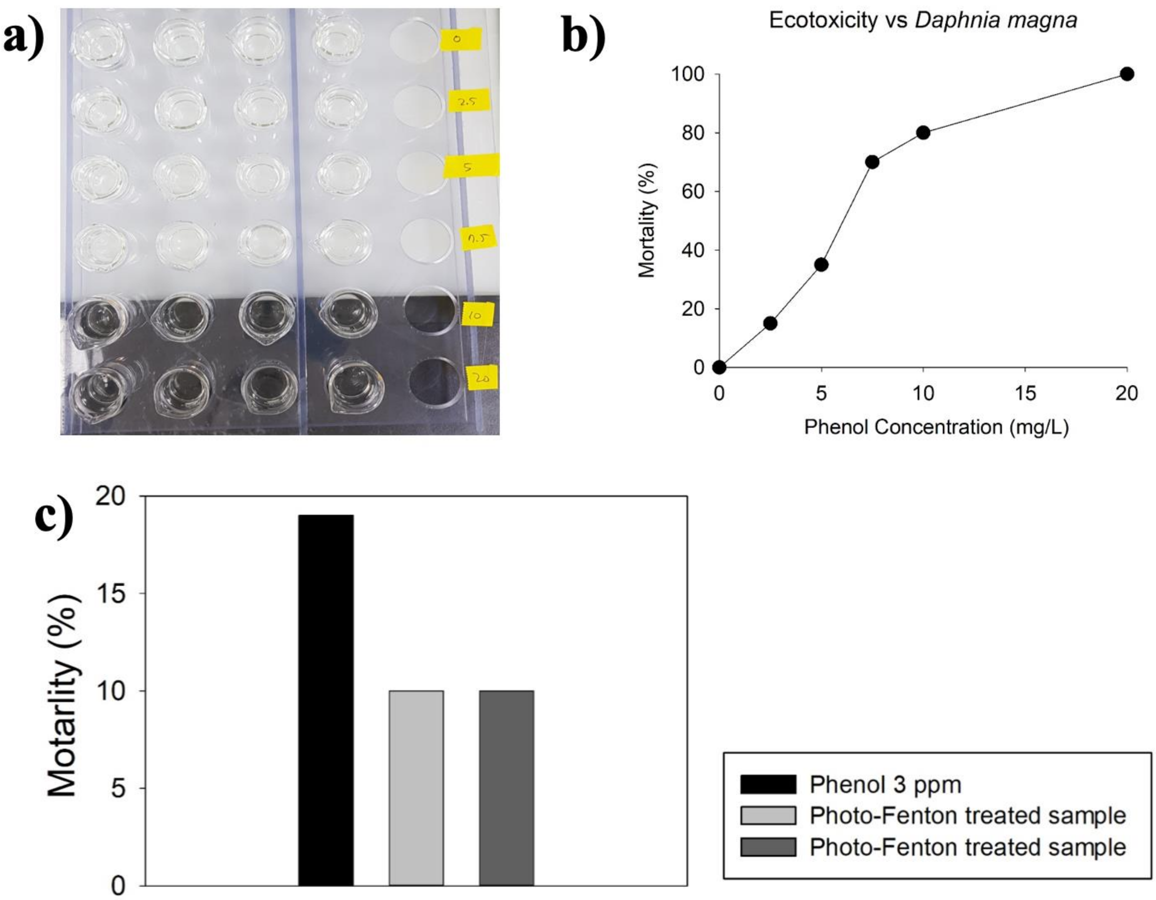

Figure S1. Ecotoxicity against *Daphnia magna*: (a) exposure of *D. magna* to different concentrations of phenol, (b) ecotoxicity of phenol against *D. magna*, and (c) ecotoxicity of phenol 3 ppm (initial sample), photo-Fenton-, and photocatalysis-treated samples

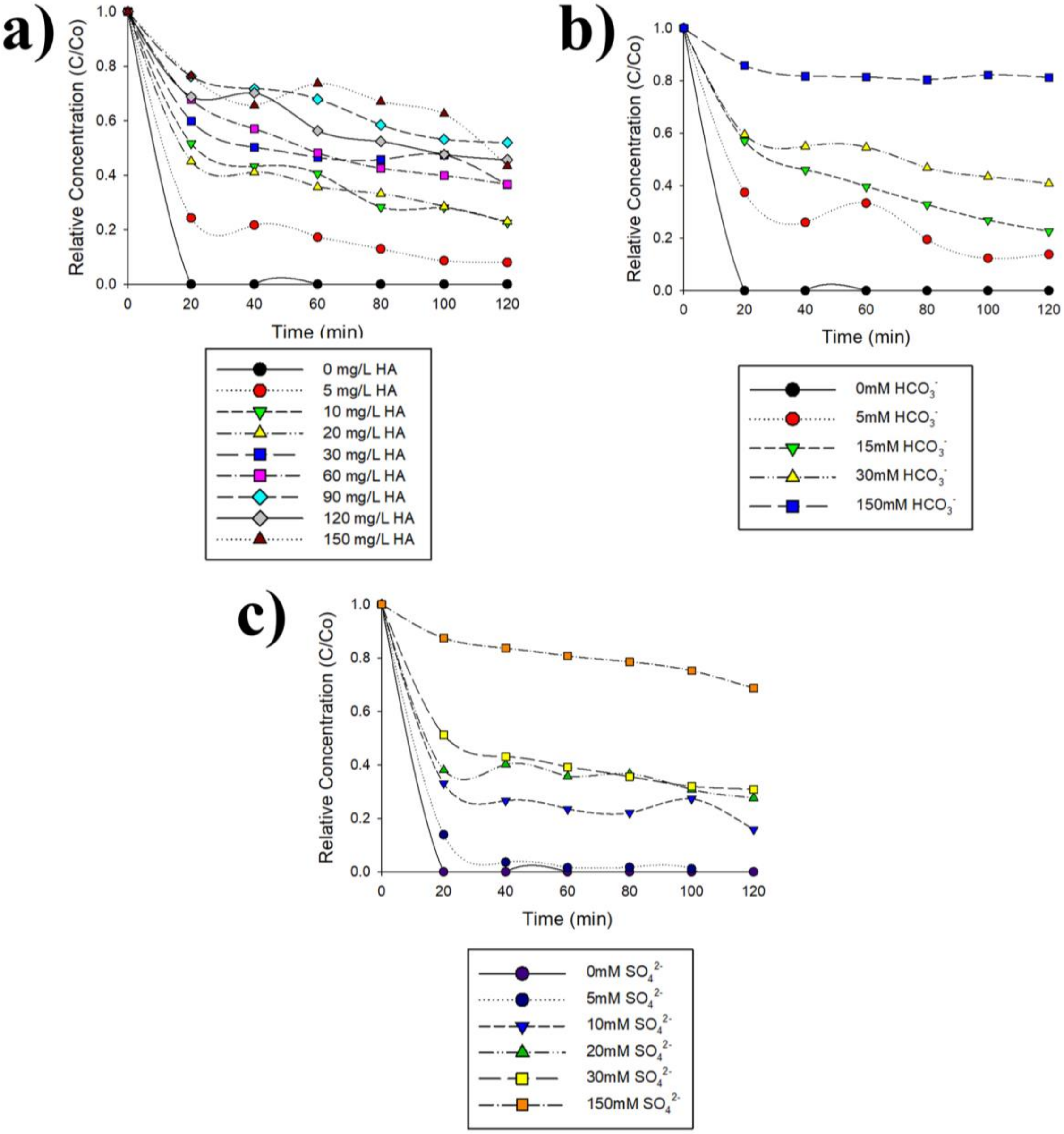

Figure S2. Effects of inhibition agents: a) HA, b) HCO_3^-, and c) SO_4^{2-} on photo-Fenton performance of MgAC-Fe_3O_4 [0.05 g]/TiO_2 hybrid nanocomposites

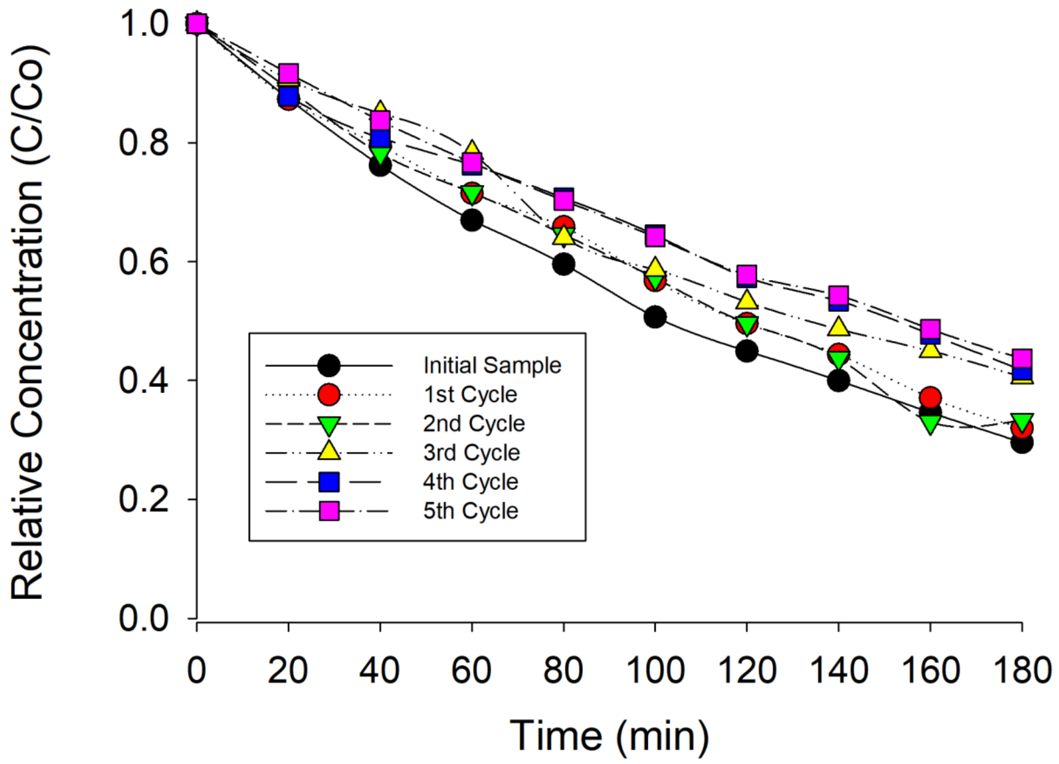

Figure S3. Recycling performances of MgAC-Fe_3O_4 [0.05 g]/TiO_2 on batch mode scale

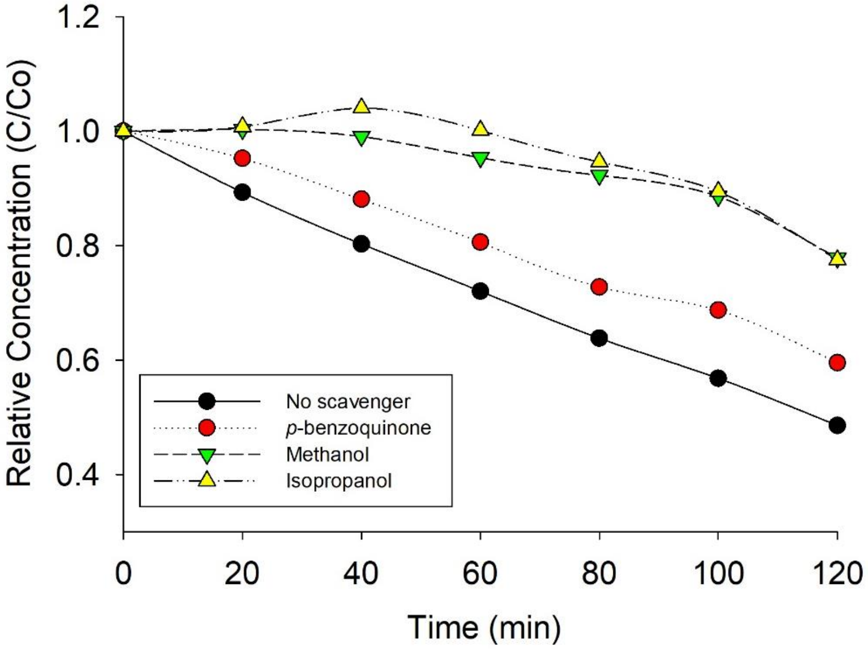

Figure S4. Photocatalytic activities of MgAC-Fe_3O_4 [0.05 g]/TiO_2 against 20 ppm MB in the presence of different reactive species: 0.1 M isopropanol, 0.1 M ethanol, and 5mM *p*-benzoquinone

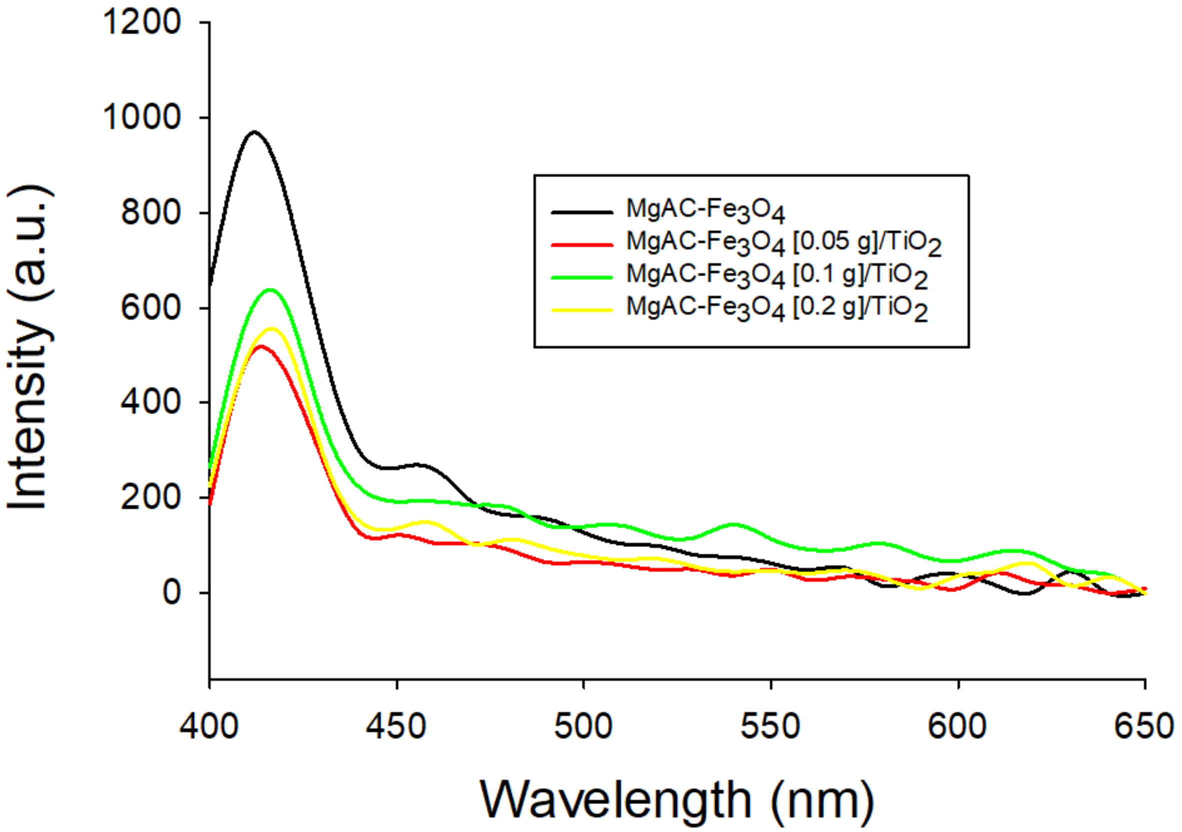

Figure S5. Photoluminescence (PL) spectra of MgAC-Fe_3O_4 and MgAC-Fe_3O_4/TiO_2 hybrid nanocomposites (λ_{ex} = 365 nm).

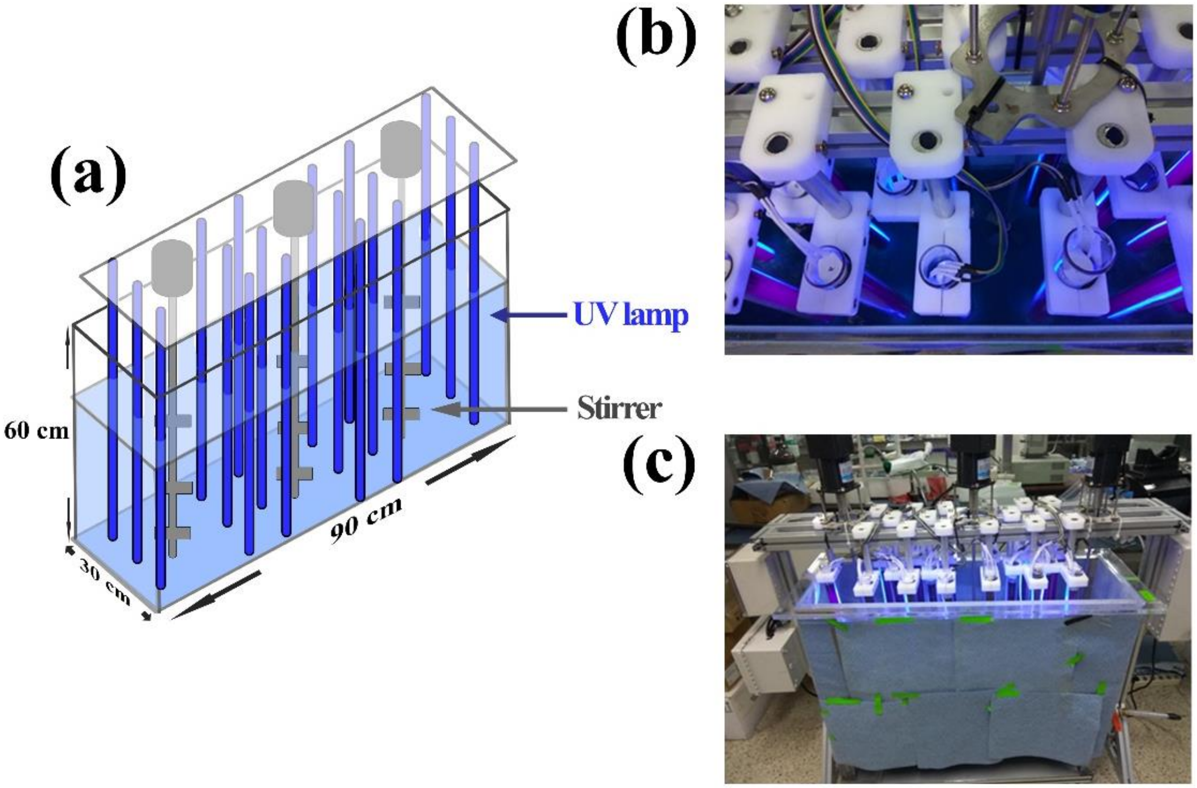

Figure S6. (a) Design of reactor for pilot-scale experiment and photos of reactor from (b) above and (c) front

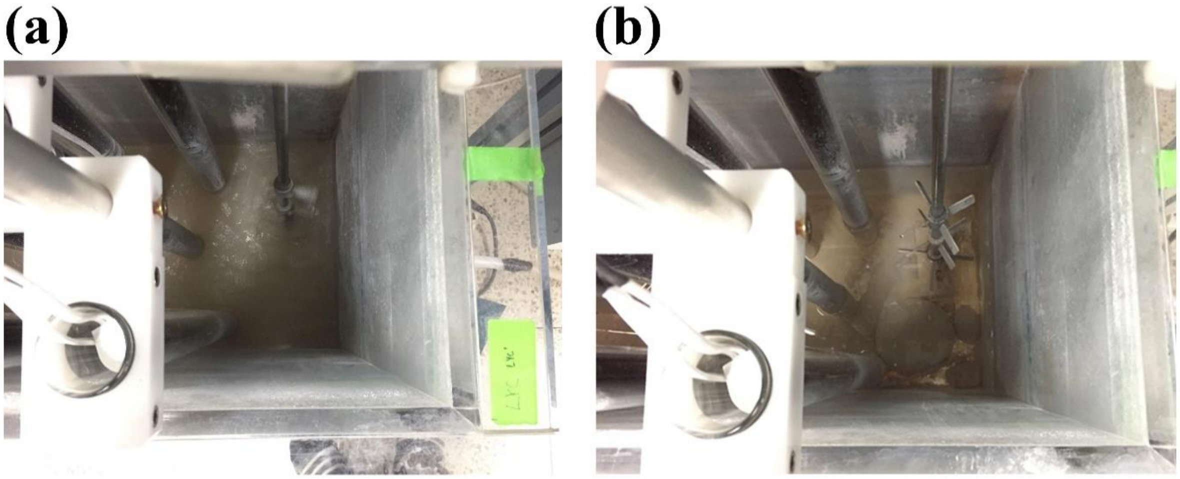

Figure S7. Photos of reactor (a) when finishing reaction and (b) after self-precipitation for 24 hours

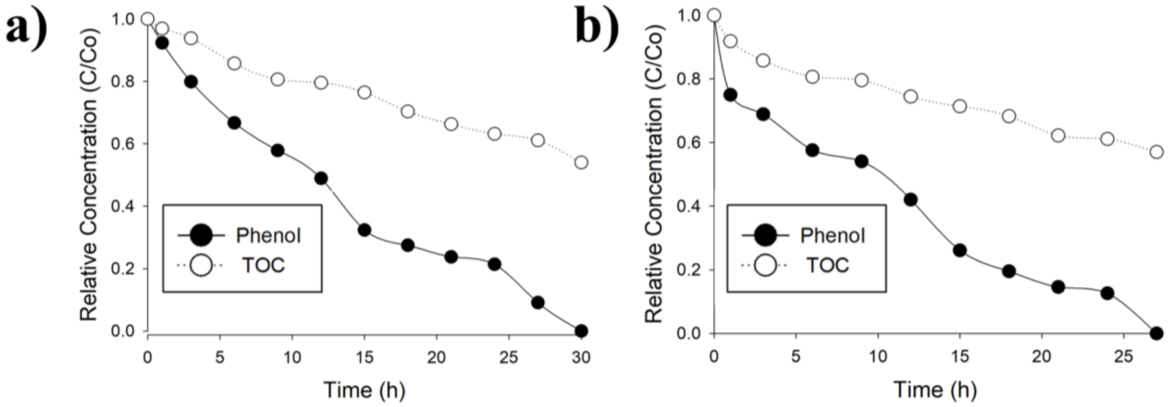

Figure S8. Phenol concentration vs. TOC concentration during (a) photo-Fenton and (b) photocatalytic reactions by MgAC-Fe_3O_4 [0.05 g]/TiO_2 on pilot scale

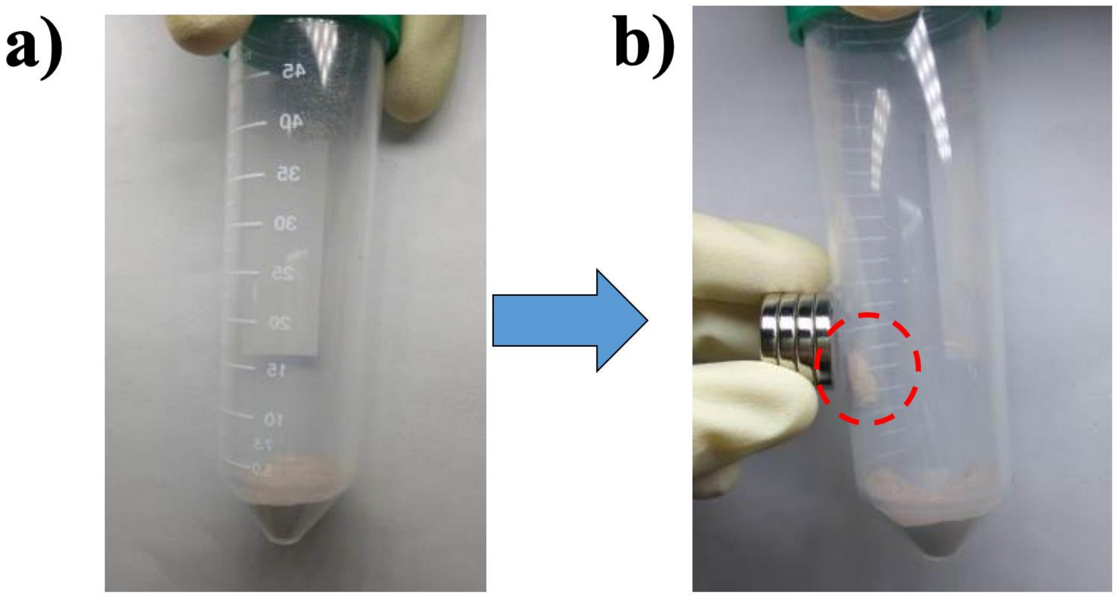

Figure S9. MgAC-Fe_3O_4 [0.05 g]/TiO_2 hybrid nanocomposites (a) before and (b) after applied magnetic field

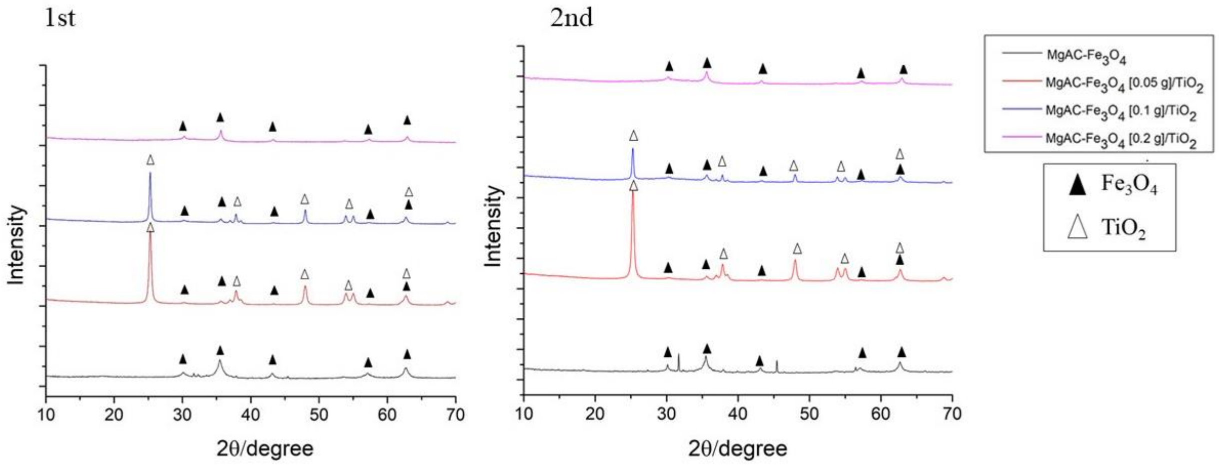

Figure S10. XRD was conducted two times to confirm the absence of TiO_2 peaks due to the excess MgAC-Fe_3O_4 loading.

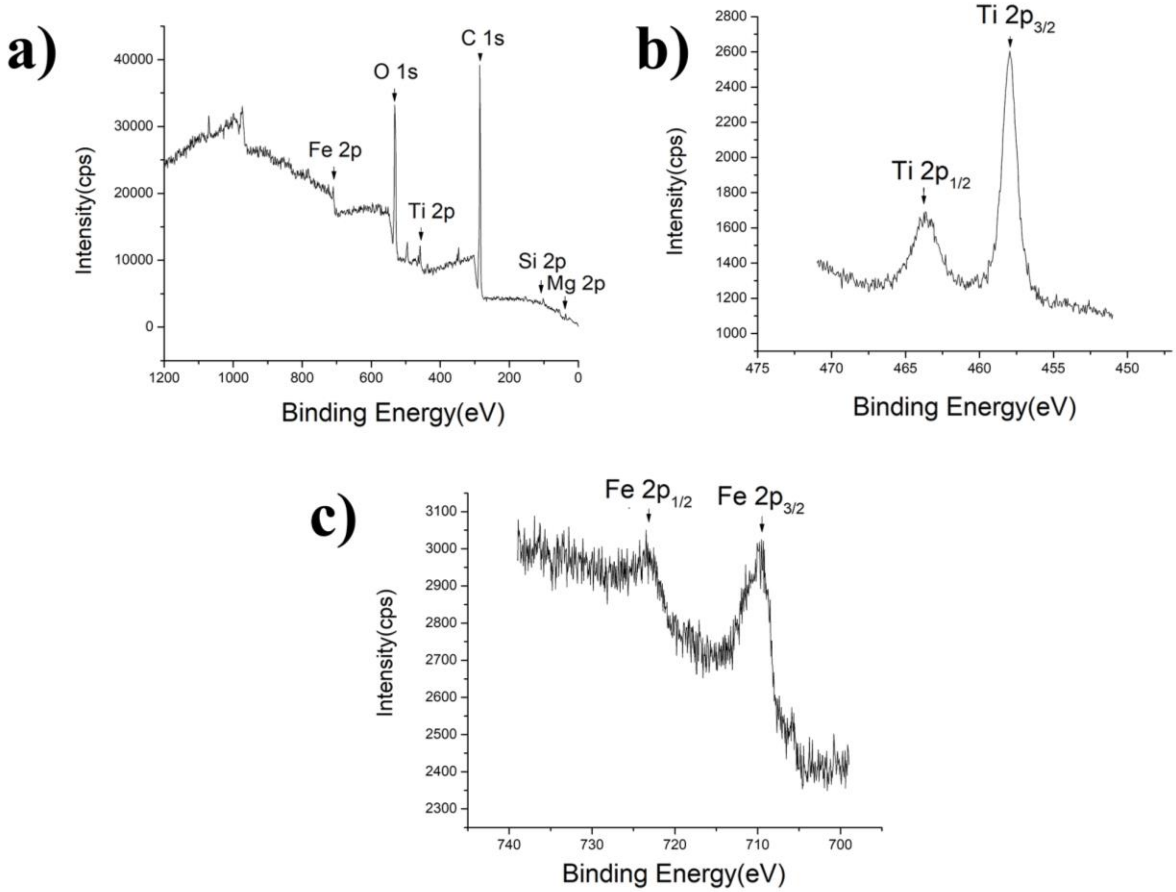

Figure S11. XPS spectra of MgAC-Fe_3O_4 [0.05 g]/TiO_2: (a) full spectra, (b) Ti 2p region, (c) Fe 2p region

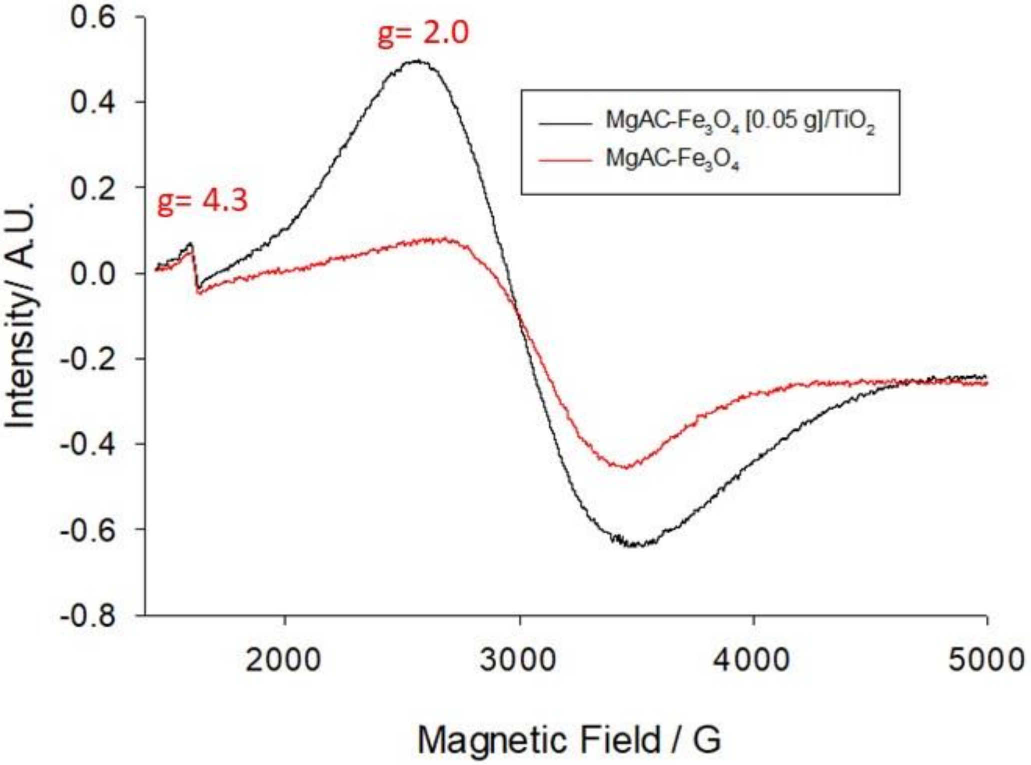

Figure S12. ESR spectra of MgAC-Fe_3O_4 and MgAC-Fe_3O_4 [0.05 g]/TiO_2

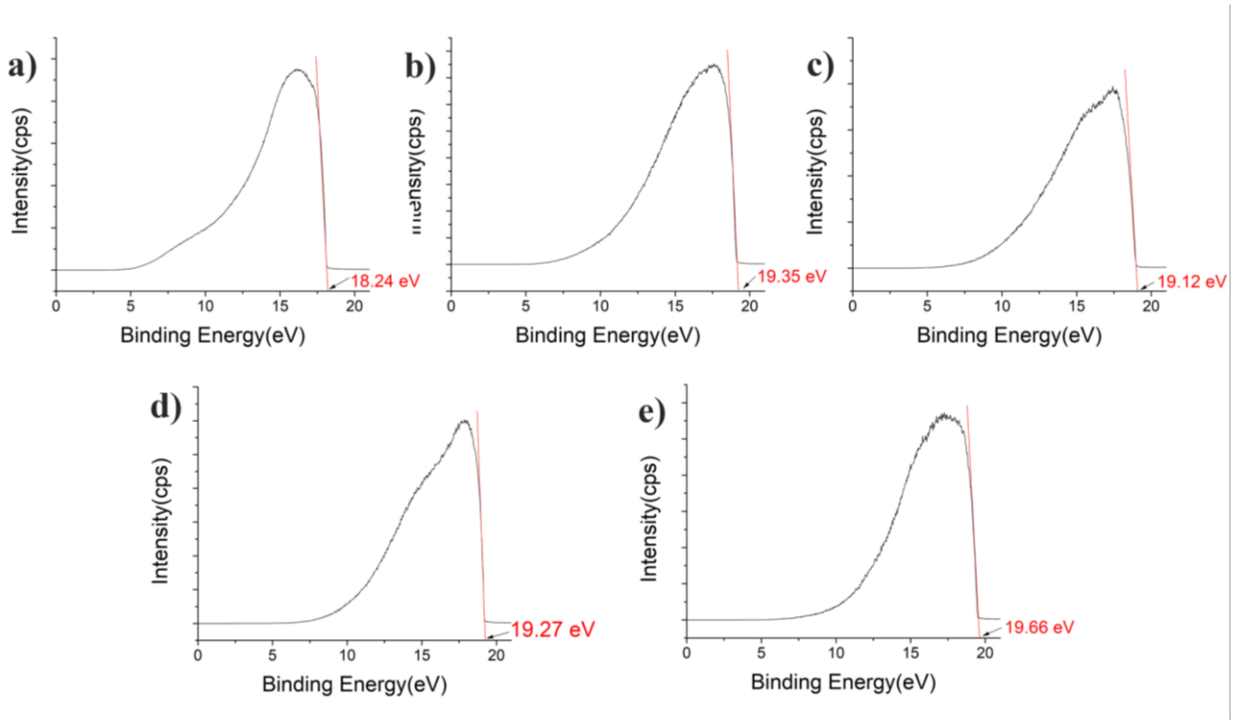

Figure S13. UPS spectra and secondary electron cutoff energies (E_{cutoff}) of (a) MgAC-Fe_3O_4, (b) MgAC-Fe_3O_4 [0.05 g]/TiO_2, (c) MgAC-Fe_3O_4 [0.1 g]/TiO_2, (d) MgAC-Fe_3O_4 [0.2 g]/TiO_2, and (e) MgAC-TiO_2

Table S1. Elemental compositions (wt. %) of as-prepared samples by X-ray fluorescence (XRF) spectrometry

**Sample**	**MgAC-Fe_3O_4 [0.05 g]/TiO_2**	**MgAC-Fe_3O_4 [0.1 g]/TiO_2**	**MgAC-Fe_3O_4 [0.2 g]/TiO_2**	**MgAC-Fe_3O_4**
MgO (%)	3.5	3.4	3.5	6.8
SiO_2 (%)	0.91	0.73	1.1	2.4
TiO_2 (%)	88.1	81.5	74.3	0.01
Fe_3O_4 (%)	7.48	14.4	21.2	90.8

Table S2. Work functions of materials in this study

Sample	Work function (eV)
MgAC-Fe_3O_4	2.98
MgAC-Fe_3O_4 [0.05 g]/TiO_2	1.87
MgAC-Fe_3O_4 [0.1 g]/TiO_2	2.10
MgAC-Fe_3O_4 [0.2 g]/TiO_2	1.98
MgAC-TiO_2	1.56

Table S3. Cost estimations

Product	Estimated Cost (USD/kg)*
MgAC	389.80
MgAC-Fe_3O_4	583.45
MgAC-TiO_2	1476.24
**MgAC-Fe_3O_4 [0.05 g]/TiO_2**	**1479.80**

*Estimated cost includes Republic of Korea industrial-electrical fees.

*Estimated cost does not include the cost of 4% H_2/Ar.
